# Preparation and Performance Study of Ultraviolet-Responsive Self-Healing Epoxy Asphalt

**DOI:** 10.3390/ma17174403

**Published:** 2024-09-06

**Authors:** Jian Wang, Maoan Wang, Shuwen Xu, Fenglei Zhang

**Affiliations:** 1 Shanxi Lixi Expressway Co., Ltd., Lvliang 033400, China; 990202300103@cqjtu.edu.cn; 2 School of Highway, Chang’an University, Xi’an 710064, China; 2022221189@chd.edu.cn (M.W.); 2022221214@chd.edu.cn (S.X.); 3 National & Local Joint Engineering Research Center of Transportation Civil Engineering Materials, Chongqing Jiaotong University, Chongqing 400074, China

**Keywords:** epoxy asphalt, UV irradiation, crack repair, photochemical reaction

## Abstract

In this study, a self-healing epoxy asphalt material was developed by incorporating coumarin groups. This material achieved microcrack self-repair under UV irradiation at 50 °C. Fluorescence microscopy observations and mechanical performance tests demonstrated significant advantages in crack filling and mechanical property recovery after repair, with the fracture toughness of the repaired epoxy asphalt reaching 69% of that in its original state. Furthermore, the synergistic effect of temperature and UV irradiation in the self-healing process enhanced the material’s durability and service life. This research offers new insights and methods for developing more durable and long-lasting self-healing asphalt materials, showcasing the great potential of smart materials in infrastructure applications.

## 1. Introduction

With the advancement of global infrastructure construction, bridge building has entered a new era, marked by the increases in the construction of long-span bridges [1]. Research indicates that one of the most effective structural forms for such bridges is the steel box girder bridge [2]. The decks of these long-span steel box girder bridges are predominantly paved with orthotropic bridge decks and asphalt mixtures [3]. The service conditions of the pavement on long-span steel bridge decks are extremely demanding, necessitating exceptionally high standards for the pavement materials [4]. Epoxy asphalt concrete, with its high strength, excellent fatigue and corrosion resistance, superior high-temperature stability, and water resistance, has emerged as a favored material [5]. Its shrinkage coefficient closely matches that of steel at low temperatures, significantly reducing temperature-induced stress [6]. Consequently, an increasing number of long-span bridges are adopting epoxy asphalt concrete as the primary material for bridge deck pavement [7].

However, due to the unique service conditions, construction techniques, and quality control requirements, the pavement on long-span steel bridge decks is more susceptible to damage compared to road pavements [8]. Among the various types of damage, pavement cracks have become a critical factor affecting the service life of bridge decks. Epoxy asphalt, the primary material for bridge deck pavement, is composed mainly of epoxy resin and base asphalt in a specific ratio. Epoxy resin, a thermosetting material, exhibits high brittleness due to its dense crosslinking and unique molecular structure [9]. This brittleness makes it prone to microcracks under external impacts. These cracks not only compromise the safety of high-speed driving and the durability of the steel bridge surface but also have adverse social impacts [10]. Cracks in the pavement allow rain and moisture to penetrate, leading to the corrosion of the steel bridge deck, which can directly impair the structural integrity of the bridge [11]. Given that stress concentrates on the surface of steel decks, fatigue cracks in pavement typically initiate at the surface and progressively propagate to the base. Therefore, study of thermosetting epoxy asphalt materials for steel bridge deck paving in terms of self-healing is needed.

Asphalt, a viscoelastic material derived from petroleum refining, inherently possesses self-healing properties [12,13]. Research by Kim et al. [14] has highlighted the importance of the morphology and size of asphalt functional groups in the healing process. Bahsin et al. [15] developed a crack-healing model that represents the time-dependent processes of wetting and internal healing. Sun et al. [16] explored the temperature-dependent crack healing process of asphalt. Additionally, Erik Schlangen [17], from Delft University of Technology, and Su Junfeng [18], from Tianjin University of Technology, have developed a self-healing method for asphalt concrete using microcapsules. This method involves encapsulating low-viscosity asphalt rejuvenators within polymer capsules and incorporating them into the asphalt concrete. When cracks form in the concrete, they rupture the capsules, releasing the rejuvenator to repair the cracks and regenerate aged asphalt.

**Figure 1:**
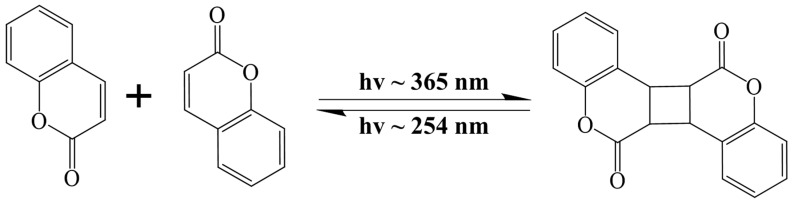
Reversible photocycloaddition process of coumarin.

Unlike conventional asphalt concrete, thermosetting epoxy asphalt does not exhibit self-healing properties after cracking [19]. There are few studies reported in the existing literature on the self-healing aspects of epoxy asphalt. Although research on the self-healing of epoxy asphalt is limited, the study of self-healing in thermosetting epoxy resin has gained significant attention [20]. The self-healing behavior of thermosetting resins is often divided into two forms: photoreversible and thermoreversible [21]. Studies have shown that reversible photocyclization reactions hold great potential for self-healing materials [22], as they enable the repair of repeated damage at the same location. This photochemical reaction is cost-effective as well as environmentally friendly and can occur under sunlight. Certain alkenes can undergo cyclodimerization to form cyclobutane structures under ultraviolet (UV) light with a wavelength exceeding 300 nm [23]. Under shorter-wavelength UV light, these cyclobutane compounds can revert to their original olefins [24]. Researchers have leveraged this reversible dimerization to develop polymer materials with self-healing capabilities [25,26]. For instance, Sanjib Banerjee et al. [27], from the University of Massachusetts Lowell, developed a star-shaped polyisobutylene containing coumarin groups, which can rapidly crosslink under 365 nm UV irradiation and undergo photolysis under 254 nm UV irradiation, thereby demonstrating excellent repeated self-repair performance. The relevant reaction equation is illustrated in Figure 1. Additionally, George P. Simon and Kei Saito, from Monash University in Australia, introduced photoreversible dimerization to enable epoxy polymers to transition from a rigid crosslinked structure to a flowable state, facilitating damage repair and re-curing of the crosslinked structure [28].

In this study, the compound 4-methylumbelliferone, containing coumarin groups, was used alongside a commercially available epoxy curing agent to develop a self-healing epoxy asphalt material that can heal under sunlight. Microcracks in the epoxy asphalt were repaired through UV irradiation at 365 nm and 254 nm. The self-healing process was observed using fluorescence microscopy (FM), the reaction mechanism was elucidated through Fourier-transform infrared spectroscopy (FTIR), and the mechanical properties of the self-healing epoxy asphalt were analyzed using a universal tensile machine (UTM). Finally, the self-healing mechanism of epoxy asphalt containing coumarin groups was thoroughly investigated.

## 2. Experimental Part

### 2.1. Experimental Materials and Equipment

The experimental reagents and materials utilized in this study are listed in Table 1, while the experimental equipment used during the preparation process is detailed in Table 2.

Both the epoxy curing agent and epoxy resin employed in this research were commercially available, with their specific technical specifications provided in Table 3 and Table 4. The epoxy resin and curing agent were both TAF brand, manufactured by Japan’s Taiyo Construction Co. (Tokyo, Japan) The characteristics of matrix asphalt (70#) measured according to JTG E20-2011 (China) are shown in Table 5.

### 2.2. Preparation of Epoxy Asphalt and Self-Healing Epoxy Asphalt

#### 2.2.1. Preparation of Epoxy Asphalt

**Figure 2:**
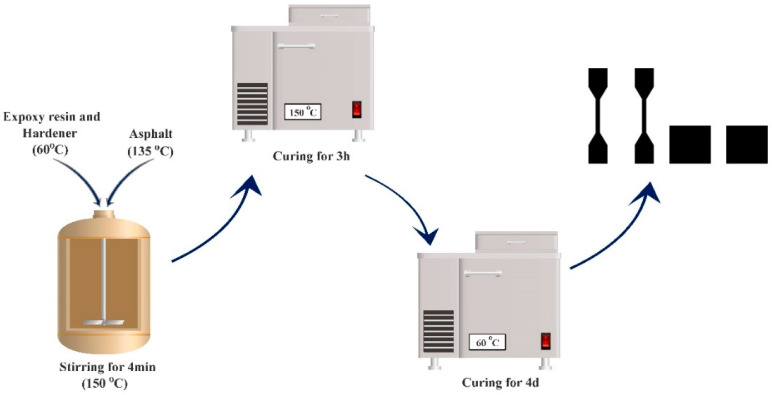
Preparation process of epoxy resin.

First, the base asphalt was heated to 135 °C. The epoxy curing agent and epoxy resin were mixed at a mass ratio of 44:56 and heated to 60 °C for subsequent use. The heated epoxy mixture and base asphalt were then combined at a mass ratio of 50:50, stirred for 4 min, and placed in an oven at 150 °C to cure for 3 h. After this, the mixture was transferred to an oven set at 60 °C for an additional 4 days of curing, followed by a day at room temperature to produce the epoxy asphalt material. The preparation process is illustrated in Figure 2.

#### 2.2.2. Preparation of Self-Healing Epoxy Asphalt

**Figure 3:**
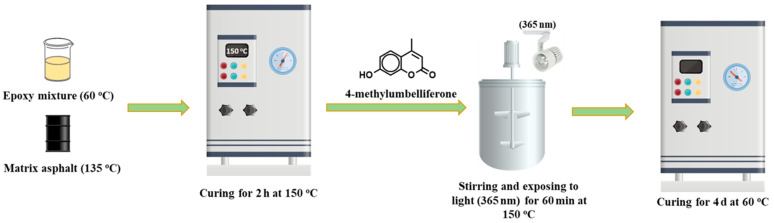
Preparation process of self-healing epoxy resin.

In a parallel process, the base asphalt was again heated to 135 °C. The epoxy curing agent and resin were mixed at a mass ratio of 44:112 and heated to 60 °C. The epoxy mixture and base asphalt were then combined at a 1:2 mass ratio, stirred for 4 min, and cured in an oven at 150 °C for 2 h. Following this, a mixture of tetramethyl umbelliferone and epoxy resin was prepared at a mass ratio of 29:112. Since no active amino groups were present in the reaction system at this stage, a Michael addition reaction was avoided. Under 365 nm UV lamp irradiation, the mixture was stirred at 150 °C for 60 min and then placed in a 60 °C oven for 4 days, followed by a day at room temperature to produce the self-healing epoxy asphalt material. This preparation process is depicted in Figure 3.

## 3. Experimental Results and Discussion

### 3.1. Microscopic Analysis

At the microscopic level, various characterization techniques were employed to analyze the structure and morphology of the epoxy asphalt, including fluorescence microscopy (FM). Through FM analysis, the distribution and morphology of epoxy within the asphalt could be visualized, providing valuable insights into the appropriate amount of modifier to use and the modifying mechanism [29]. During the high-temperature curing process, the epoxy asphalt was dropped onto a glass slide. The epoxy asphalt samples were subjected to both high-temperature curing and low-temperature curing processes, and then the cured epoxy asphalt was observed under a fluorescent microscope. Each sample was examined five times at different locations using a microscope manufactured by Muou Technology Co. (Sanya, China), equipped with a self-contained fluorescent lamp. Fourier-transform infrared spectroscopy (FTIR) spectroscopy was performed using a Nicolet 5700 FTIR spectrometer (USA Thero Electron Corporation, Waltham, MA, USA), using an Attenuated Total Reflection (ATR) attachment for the experiments in a range of 4000–650 cm^−1^ at a resolution of 4 cm^−1^ and 32 scans. FTIR was utilized to characterize the functional groups, allowing the identification of the chemical reactions between the modifier and asphalt, thereby offering a deeper understanding of the reaction mechanism. By correlating these microscopic analyses with the macroscopic performance of the asphalt, this study aimed to elucidate the mechanism of polymer-modified asphalt and provide a theoretical foundation for future developments in this area.

#### 3.1.1. FTIR Spectroscopy

**Figure 4:**
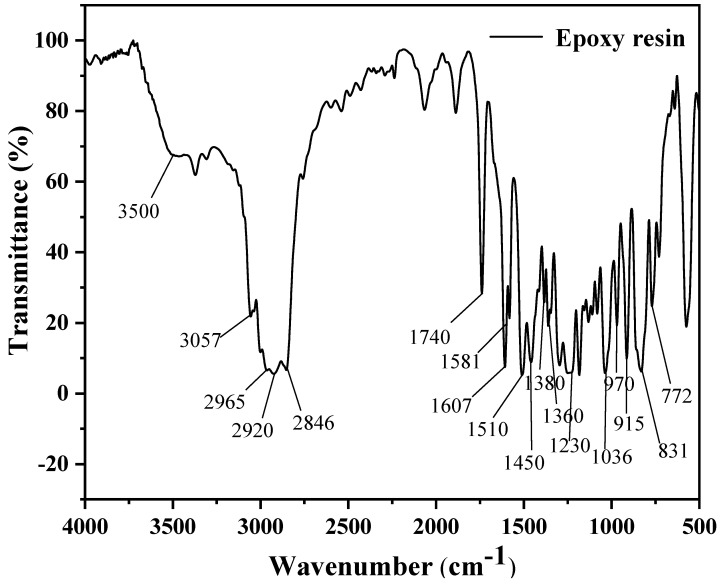
FTIR spectrum of epoxy resin.

The FTIR analysis results of the epoxy resin are shown in Figure 4.

The broad peak at 3500 cm^−1^ corresponds to the stretching vibration of the hydroxyl O-H bond, while the peak at 3057 cm^−1^ indicates the stretching vibration of the C-H bond on the ethylene oxide ring. The stretching vibration peaks of CH_2_ and CH in aromatic and aliphatic compounds are observed between 2965 cm^−1^ and 2846 cm^−1^. The peak at 1740 cm^−1^ is attributed to the stretching vibration of the C=O bond, and the strong absorption peak at 1607 cm^−1^ corresponds to the stretching vibration of the C=C bond on the aromatic ring. Peaks at 1581 cm^−1^ and 1510 cm^−1^ are associated with the stretching vibrations of the benzene ring in place of the C-C bond. The peak at 1450 cm^−1^ is inferred to represent the in-plane bending vibration of the C-H bond. Strong absorption peaks at 1380 cm^−1^ and 1360 cm^−1^ are characteristic of the symmetrical deformation vibrations of the dimethyl groups in bisphenol A epoxy resin. The aromatic ether bond stretching vibration peak is found at 1230 cm^−1^, and the fatty ether C-O-C bond peak appears at 1036 cm^−1^. The peak at 970 cm^−1^ is due to the out-of-plane bending vibration of the C-H bond, while the peak at 915 cm^−1^ is caused by the stretching vibration of the C-O bond in the oxirane group. The broad peak at 831 cm^−1^ is attributed to the overlapping stretching vibration peak of the C-O-C bond in the ethylene oxide functional group and the p-substituted peak of the benzene ring. The peak at 721 cm^−1^ is ascribed to the rocking vibration of the CH_2_ group when the carbon chain contains four or more carbon atoms.

**Figure 5:**
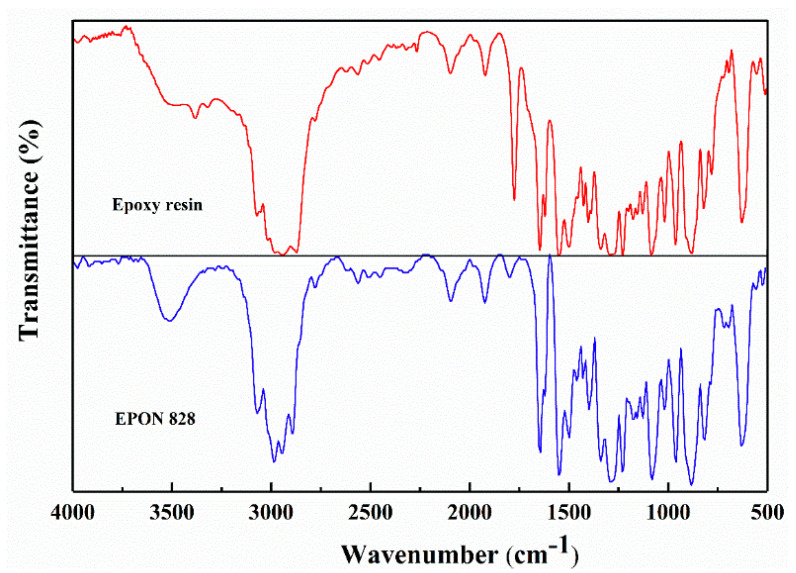
Infrared spectrum matching of epoxy resin.

The infrared spectrum of the epoxy resin was matched using OMNIC 9.0 software, as shown in Figure 5.

A comparison revealed a 92.14% similarity between the commercially available epoxy resin and bisphenol A epoxy resin (EPON 828). By analyzing the functional groups presented in Figure 4, it can be concluded that the epoxy resin used in this study was a typical bisphenol A epoxy resin.

**Figure 6:**
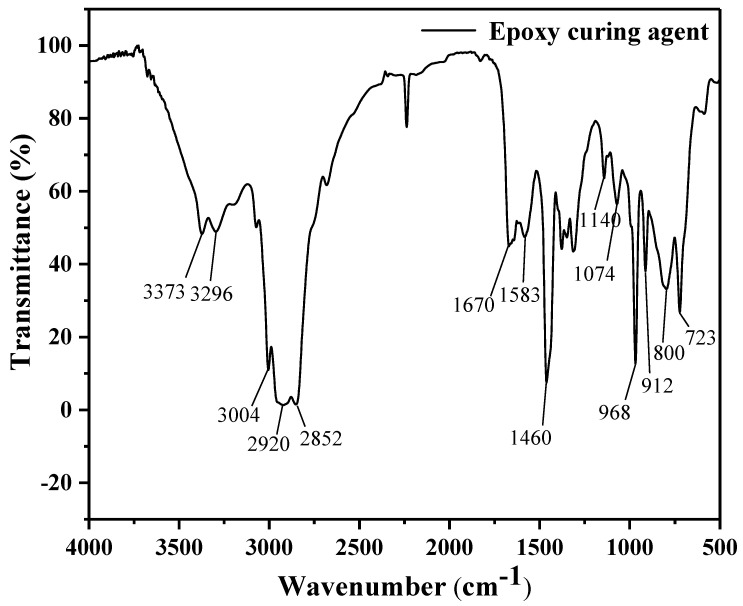
FTIR spectrum of epoxy curing agent.

The FTIR analysis results for the epoxy curing agent are presented in Figure 6. 

In Figure 6, the spectrum shows symmetric and asymmetric stretching vibration peaks of the primary amine at 3373 cm^−1^ and 3296 cm^−1^, respectively. The unsaturated C-H bond stretching vibration peak in olefin is observed at 3004 cm^−1^, while the saturated C-H bond stretching vibration peaks appear at 2920 cm^−1^ and 2852 cm^−1^. The peak at 1670 cm^−1^ corresponds to the C=C bond stretching vibration, and the peak at 1583 cm^−1^ is attributed to the N-H bond’s bending vibration. The C-H bond’s stretching vibration peak is found at 1460 cm^−1^, while the peak at 1140 cm^−1^ represents the C-N bond’s stretching vibration in aliphatic hydrocarbons. Peaks at 1074 cm^−1^ are attributed to the scissoring vibration of the N-H bond. The peak at 800 cm^−1^ is associated with the out-of-plane bending vibration of the N-H bond, while the peak at 968 cm^−1^ is inferred to be the out-of-plane deformation vibration of the trans-1,4-CH=CH- group. The peak at 912 cm^−1^ is linked to the out-of-plane deformation vibration of the 1,2-CH=CH_2_ group, and the peak at 723 cm^−1^ corresponds to the rocking vibration of the CH_2_ group when the carbon chain contains four or more carbon atoms.

**Figure 7:**
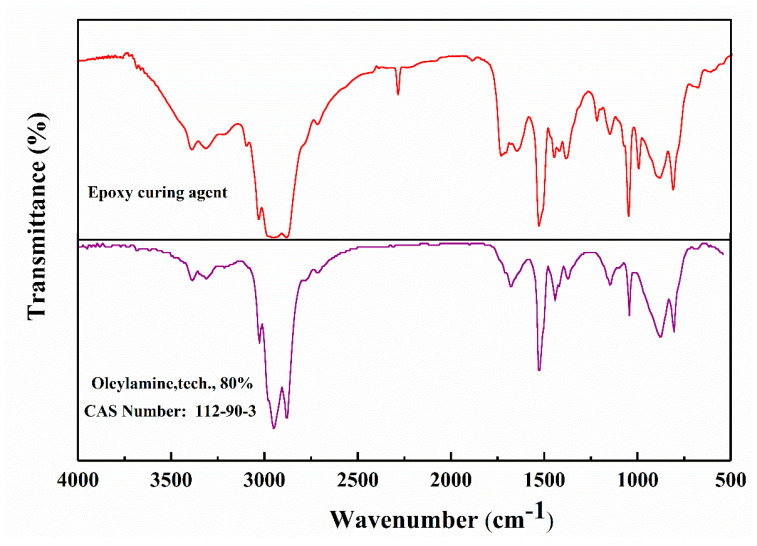
Infrared spectrum matching of epoxy curing agent.

The infrared spectrum of the epoxy curing agent was matched using OMNIC software, as shown in Figure 7. 

The analysis revealed an 85.62% similarity between the epoxy curing agent and oleylamine. A comparison of the functional groups in the infrared spectrum confirmed that the primary component of the epoxy curing agent was oleylamine.

**Figure 8:**
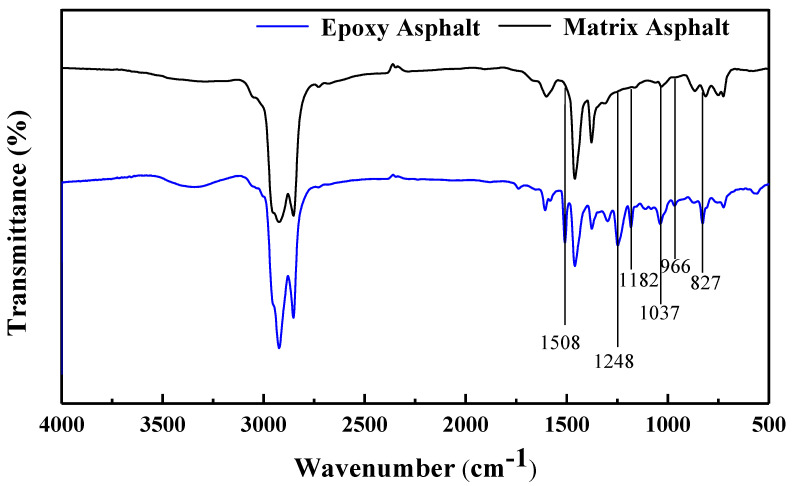
FTIR spectrum of epoxy asphalt and matrix asphalt.

Finally, the infrared spectrum analysis of the epoxy asphalt and base asphalt is shown in Figure 8.

As shown in Figure 8, the disappearance of the infrared peak associated with the N-H bond confirms that the epoxy resin had fully cured. The peak at 1508 cm^−1^ corresponds to the skeletal vibration of the para-substituted benzene ring, while the peak at 1248 cm^−1^ is attributed to the stretching vibration of the aromatic ether bond. A new stretching vibration peak of the aliphatic amine C-N bond appears at 1182 cm^−1^, and the stretching vibration absorption peak of the aliphatic ether C-O-C bond is observed at 1037 cm^−1^. The peak at 966 cm^−1^ corresponds to the absorption of the C=C bond, and the peak at 827 cm^−1^ is the para-substituted peak of the benzene ring. These findings indicate that the epoxy resin within the epoxy asphalt had fully cured.

#### 3.1.2. Fluorescence Microanalysis

**Figure 9:**
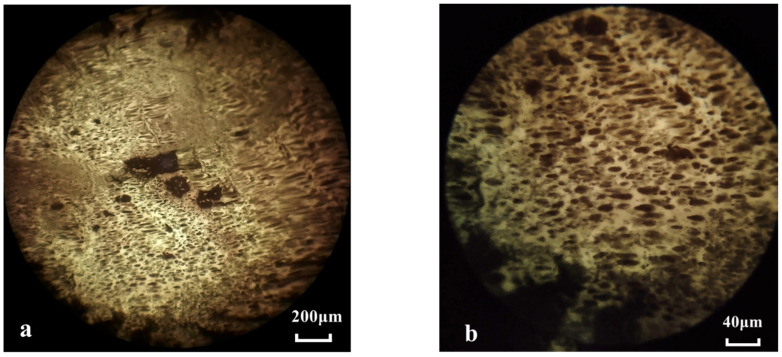
Fluorescence microscopy of cured epoxy asphalt: (**a**) 100×, (**b**) 250×.

The fully cured epoxy asphalt was placed on a glass slide and then observed under a fluorescence microscope, as shown in Figure 9.

In Figure 9, under fluorescence conditions, the epoxy resin appears yellow, while the asphalt phase appears black. The fluorescence micrograph of the epoxy asphalt shows that the epoxy resin forms a cross-linked network structure within the asphalt. This structure prevents the asphalt from flowing at high temperatures and improves its crack resistance at low temperatures, fundamentally altering the thermoplastic behavior of the asphalt and imparting excellent performance.

**Figure 10:**
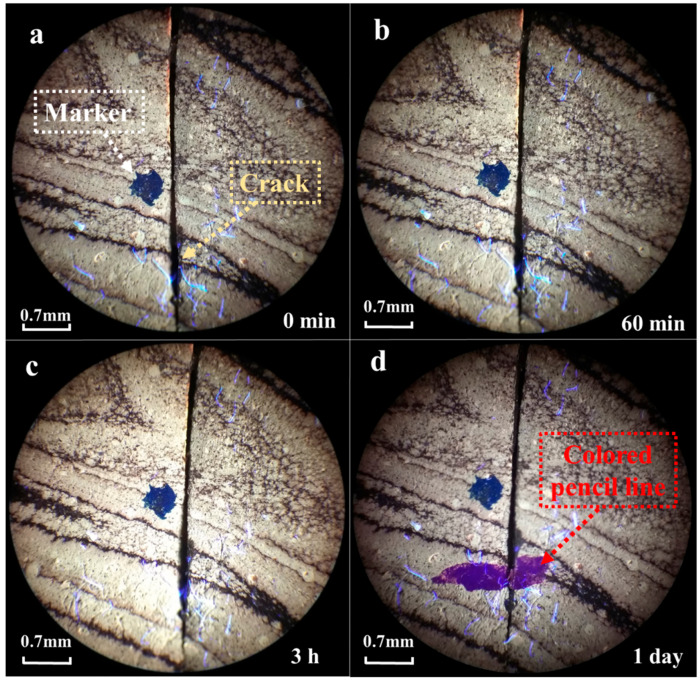
Observation of epoxy asphalt cracking at 30 °C: (**a**) 0 min, (**b**) 60 min, (**c**) 3 h, (**d**) 1 day.

To explore the self-healing effect of epoxy asphalt cracks using fluorescence microscopy, a blade was used to create a crack approximately 0.1 mm in size on the epoxy asphalt. The healing of the crack was then observed at different temperatures. The healing condition of the epoxy asphalt crack at 30 °C is shown in Figure 10.

As shown in Figure 10, after 3 h at 30 °C, the crack in the epoxy asphalt did not significantly heal, as illustrated in Figure 10b,c. After leaving it at room temperature for one day, a colored pen was used to mark the crack, and a noticeable groove appeared at the mark, indicating that the crack remained and had not healed. This suggests that the crack in the epoxy asphalt could not self-heal at 30 °C.

**Figure 11:**
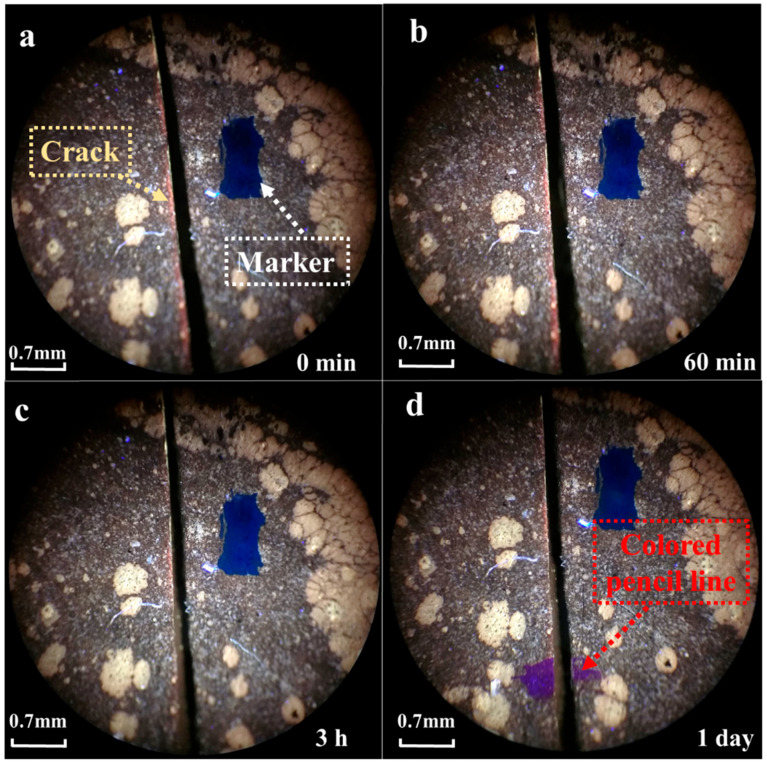
Observation of epoxy asphalt cracking at 40 °C: (**a**) 0 min, (**b**) 60 min, (**c**) 3 h, (**d**) 1 day.

Next, the self-healing of a 0.1 mm crack in epoxy asphalt was observed at 40 °C. The healing condition is shown in Figure 11. 

At 40 °C, the healing condition of the epoxy asphalt crack was similar to that at 30 °C. After 3 h, the crack still did not significantly heal. After leaving it at room temperature for one day and marking the crack with a colored pen, the groove caused by the crack was still visible, indicating that the crack in the epoxy asphalt could not self-heal at 40 °C.

**Figure 12:**
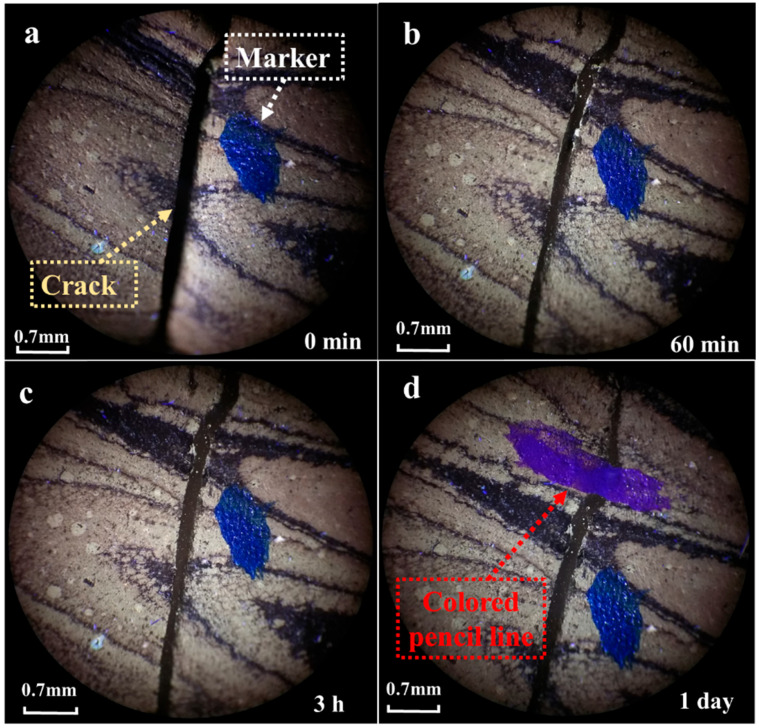
Observation of epoxy asphalt cracking at 50 °C, (**a**) 0 min, (**b**) 60 min, (**c)** 3 h, (**d**) 1 day.

The self-healing of a 0.1 mm crack in epoxy asphalt was then observed at 50 °C. The healing condition is shown in Figure 12. 

At 50 °C, it was observed that after the epoxy asphalt was left for 60 min, the crack showed a clear trend of healing compared to the initial crack. After the epoxy asphalt was placed at 50 °C for 3 h, the shape and width of the crack did not change significantly compared to the condition after 60 min. This was consistent with the condition observed at 50 °C for 60 min. After leaving it at room temperature for one day, the crack was marked with a colored pen, and the line did not break at the crack, indicating that the crack had healed. This was similar to the healing condition at 50 °C. The black areas represent the asphalt phase, while the yellow areas represent the cured epoxy resin phase. From this, the following conclusion was drawn regarding the healing condition of epoxy asphalt: For thermosetting epoxy asphalt, when the temperature approaches or reaches the softening point of the asphalt, microcracks in the epoxy asphalt can be filled as the asphalt phase flows due to the disruption of the epoxy resin phase. However, this self-healing cannot restore the material to its pre-crack condition, as the asphalt phase merely fills the crack.

**Figure 13:**
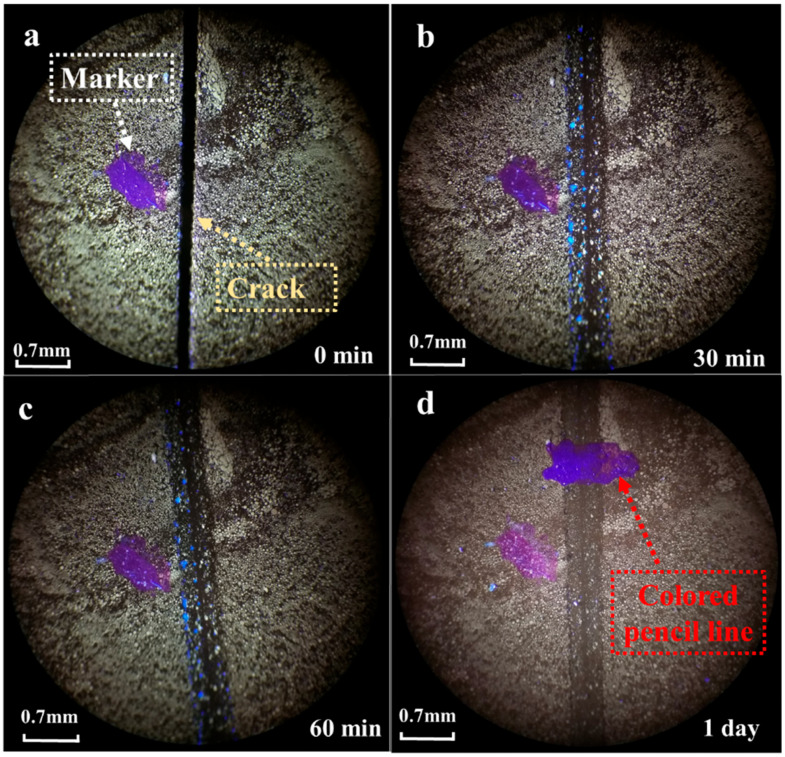
Observation of epoxy asphalt cracking at 50 °C: (**a**) 0 min, (**b**) 60 min, (**c**) 3 h, (**d**) 1 day.

Therefore, a temperature of 50 °C was selected to observe the cracks in the self-healing epoxy asphalt. The cracked self-healing epoxy asphalt was placed under 254 nm and 365 nm UV lamps while maintaining a temperature of 50 °C, as shown in Figure 13.

At 50 °C, after 30 min of UV irradiation, fluorescence microscopy observation revealed blue fluorescent material at the edges of the crack, which was speculated to be the coumarin group. Yellow fluorescent epoxy resin appeared inside the crack. After 60 min of irradiation, the amount of epoxy resin inside the crack gradually increased. After being left at room temperature for one day, the fluorescent material disappeared, and when a colored pen was used to mark the crack, it was found that the crack had disappeared, although the scratch made by the blade remained. The crack was filled with yellow epoxy resin, indicating that the asphalt in the crack had been fully encapsulated by the epoxy resin.

### 3.2. Macroscopic Performance Analysis

#### 3.2.1. Viscosity Characteristics

**Figure 14:**
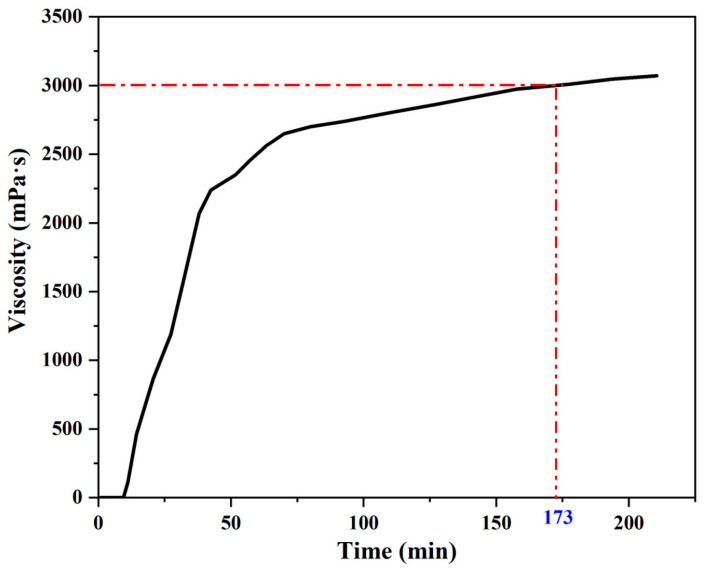
Variation in viscosity with time during curing of epoxy asphalt at 135 °C.

In accordance with the testing method specified in the AASHTO M320 standard, specifically T316, the rotational viscosity of the asphalt binder at 135 °C should be no more than 3000 mPa·s. Hence, the time required to reach a viscosity of 3000 mPa·s for the epoxy asphalt, as depicted in Figure 14, needed to be determined. The viscometer employed in this case was the DV2T rotational viscometer manufactured by Brookfield (USA), and the accessory utilized is a Thermosel heater (USA).

As shown in Figure 14, at the beginning of the curing reaction of epoxy asphalt, the viscosity changed rapidly within 60 min, and the viscosity grew slowly after the curing reaction time exceeded 70 min. The viscosity profile at 135 °C increased to 3000 mPa·s in 173 min, which adequately satisfied the need for a reserved time.

#### 3.2.2. Tensile Test Analysis

**Figure 15:**
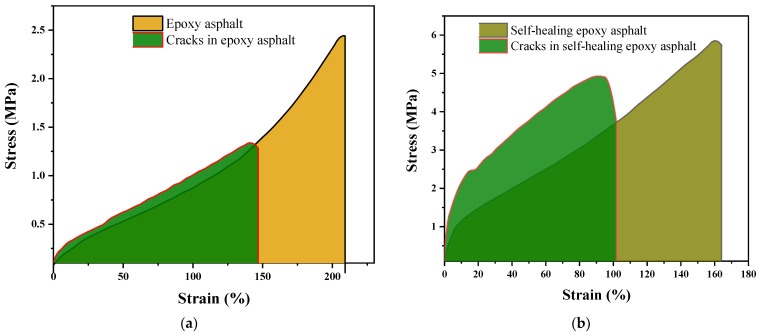
Tensile test results after fracture and healing of ordinary epoxy asphalt and self-healing epoxy asphalt. (**a**) Tensile test results of ordinary epoxy asphalt before and after healing; (**b**) Tensile test results of self-healing epoxy asphalt before and after healing.

Ordinary epoxy asphalt and the epoxy asphalt with added 4-methylumbelliferone were molded into the dumbbell shape shown in Figure 2. The middle of each specimen was then cut with a blade. These specimens were subsequently subjected to healing at 50 °C. After healing, both types of epoxy asphalt were tested in a direct tensile test at a temperature of 23 °C and a tensile rate of 500 mm/min according to ASTM D638-2010 [30] (Type IV). Tensile testing was performed using a UTM-6503 universal testing machine (Sansi Zongheng Co., Ltd., Shenzhen, China). Three standard specimens were prepared for each sample, and the mean and standard deviation were calculated based on the results of these three tests. The test results are shown in Figure 15. The test results from the figure are summarized in Table 6.

As shown in Table 6, after the fracture recovery of regular epoxy asphalt, the thermosetting epoxy resin inside it could not recover, resulting in fracture healing that was primarily due to the healing of the asphalt matrix phase. This led to a decline in mechanical properties after fracture healing. Specifically, the fracture toughness of the regular epoxy asphalt after healing was only 53% of its pre-fracture toughness, the fracture stress was 55% of its pre-fracture stress, and the fracture strain was 68% of its pre-fracture strain. In contrast, for the self-healing epoxy asphalt, the self-healing process was accompanied by the healing of the internal epoxy resin, so the decline in mechanical properties after fracture healing was less significant compared to that of the regular epoxy asphalt. After healing, the fracture toughness of the self-healing epoxy asphalt was 69% of its pre-fracture toughness, the fracture stress was 84% of its pre-fracture stress, and the fracture strain was 62% of its pre-fracture strain.

## 4. Analysis of the Self-Healing Mechanism

**Figure 16:**
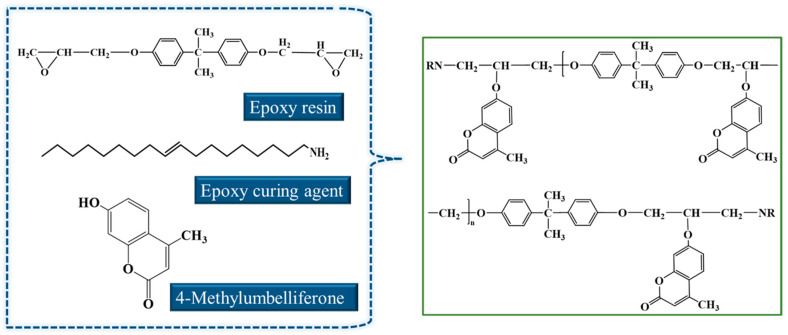
Curing mechanism of self-healing epoxy asphalt.

By analyzing the reaction of the amine-cured epoxy resin used in this study, and then adding tetramethylpiperidone, the reaction equation of the self-healing epoxy resin was determined, as shown in Figure 16.

**Figure 17:**
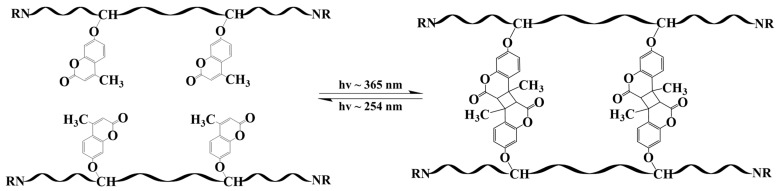
Reversible photochemical cyclization enables self-healing of epoxy asphalt.

As shown in Figure 16, the reaction of the primary amine-cured epoxy resin was widely confirmed. When 4-methylumbelliferone is added, its hydroxyl groups can further react with epoxy groups to open the ring, grafting 4-methylumbelliferone into the structure and forming a curing reaction involving 4-methylumbelliferone. The overall reaction can then undergo a reversible photochemical cyclization between 4-methylumbelliferone groups when exposed to two different wavelengths of light. This process enables the self-healing of thermosetting epoxy asphalt at crack locations. The reaction mechanism is illustrated in Figure 17.

## 5. Conclusions

1. In this study, an epoxy asphalt material containing coumarin groups was prepared, which could achieve self-healing of microcracks under UV irradiation at 50 °C.
2. The fluorescence microscopy observations showed that during the healing process, the cracks gradually filled in and eventually disappeared, indicating that the self-healing material exhibited a significant healing effect at the crack locations.
3. The mechanical performance test results indicated that the self-healing epoxy asphalt experienced minimal degradation in mechanical properties after healing, with the post-healing fracture toughness maintaining approximately 69% of that of the initial state, demonstrating superior performance compared to conventional epoxy asphalt.

In this paper, the photoreversible self-healing phenomenon of thermosetting epoxy asphalt was investigated in detail, and in future work, we hope that this material will be applied in epoxy asphalt mixtures in order to further investigate its road performance and the effect of pavement healing.

## Figures and Tables

**Table 1:** Experimental reagents.

Material	Specifications	Manufacturer
4-Methylumbelliferone	98% (Analytical purity)	Shanghai Aladdin Biochemical Technology Co., Ltd. Shanghai China
Glycerol	99% (Analytical purity)	Shanghai Aladdin Biochemical Technology Co., Ltd. Shanghai China
Talc	800 mesh	Sinopharm Chemical Reagent Co., Ltd. Beijing China
Potassium bromide	99.95% (Analytical purity)	Sinopharm Chemical Reagent Co., Ltd. Beijing China

**Table 2:** Equipment used in this study.

Device Name	Device Model	Manufacturer
Collector thermostatically heated magnetic stirrer	DF-102S	Gongyi City to China Instrument Co., Ltd. Gongyi China
Constant-temperature blast-drying oven	LABOROTA 4000	Shanghai Danding International Trade Co., Ltd. Shanghai China
Ultraviolet analyzer	ZNHW	Shanghai Lingke Development Co., Ltd. Shanghai China

**Table 3:** Technical index requirements of epoxy resin.

Type	Technical Indicators	Technical Requirement	Result
Epoxy resin	Viscosity (25 °C/mPa·s)	10–12.5	11.2
	Density (25)/g·cm^−3^	1.0–1.2	1.1
	Appearance	Transparent liquid	-

**Table 4:** Index requirements of epoxy curing agent.

Type	Technical Indicators	Technical Requirement	Result
Epoxy curing agent	Viscosity (25 °C/mPa·s)	8–10	9
	Density (25)/g·cm^−3^	0.75–1.0	0.85
	Appearance	Light yellow liquid	-

**Table 5:** Basic parameters of base asphalt (70#).

Technical Indicators	Prescribed Value	Result
Penetration (25 °C, 0.1 mm)	60–80	70.5
Ductility (5 cm/min) 10 °C (cm)	≥25	90.3
Softening point (°C)	≥45	55

**Table 6:** Summary of tensile test results after fracture and healing of ordinary epoxy asphalt and self-healing epoxy asphalt.

Category	Ordinary Epoxy Asphalt	Ordinary Epoxy Asphalt with Healing	Self-Healing Epoxy Asphalt	Self-Healing Epoxy Asphalt with Healing
Fracture Stress (MPa)	2.44 ± 0.13	1.34 ± 0.09	5.87 ± 0.21	4.92 ± 0.30
Fracture Strain	2.09 ± 0.20	1.42 ± 0.11	1.64 ± 0.18	1.01 ± 0.27
Fracture Toughness (J/m^3^)	2.19 ± 0.14	1.17 ± 0.15	5.32 ± 0.25	3.67 ± 0.37

## Data Availability

The data are available from the corresponding author upon reasonable request.
